# Knowledge mapping of the relationship between norepinephrine and memory: a bibliometric analysis

**DOI:** 10.3389/fendo.2023.1242643

**Published:** 2023-10-25

**Authors:** Qi Song, Yaqian Tan

**Affiliations:** ^1^ Department of Pharmacy, Affiliated Cancer Hospital & Institute of Guangzhou Medical University, Guangzhou, China; ^2^ Department of Pharmacy, The Affiliated Brain Hospital of Guangzhou Medical University, Guangzhou, China

**Keywords:** bibliometric analysis, visualization, norepinephrine, memory, VOSviewer, Citespace, R-*bibliometrix*

## Abstract

**Introduction:**

Memory is a fundamental cognitive function for successful interactions with a complex environment. Norepinephrine (NE) is an essential component of catecholamine induced by emotional arousal, and numerous studies have demonstrated that NE is a key regulator in memory enhancement. We therefore conducted a bibliometric analysis to represent the knowledge pattern of the literature on the theme of NE-memory relationship.

**Methods:**

The WOSCC database was selected to extract literature published during 2003-2022. The collected data of annual production, global cooperation, research structure and hotspots were analyzed and visualized.

**Results:**

Our results showed that research on the links between NE and memory displayed a considerable development trend over the last two decades. The USA had a leading position in terms of scientific outputs and collaborations. Meanwhile, University of California Irvine contributed the most publications. Benno Roozendaal and James McGaugh were the most prolific authors in this field, and *Neurobiology of Learning and Memory* had the highest number of publications on this topic. The research emphasis has evolved from memory-related diseases and brain regions to neural mechanisms for different types of memory at neural circuit levels.

**Conclusion:**

Our bibliometric analysis systematically analyzed the literature on the links between NE and memory from a bibliometric perspective. The demonstrated results of the knowledge mapping would provide valuable insights into the global research landscape.

## Introduction

Memory is a fundamental cognitive function for successful interactions with a complex environment (1, 2), and aberrant memory processes are at the core of several cognitive disorders, such as depression, Alzheimer’s disease, and post-traumatic stress disorder (PTSD) (3–12). In fact, our memories are not always retained to the same degree, and events under emotionally arousing conditions are generally better remembered (13). Emotional arousal can induce the rapid release of catecholamines, which include dopamine (DA), epinephrine, and norepinephrine (NE) (14–16). Numerous studies have suggested that NE contributes significantly during memory consolidation (17–23), as well as memory encoding and retrieval (24–26). Additionally, NE plays a major role in coordinating the memory-enhancing effects of other stress hormones during emotional memories, and the amygdala plays a pivotal role under emotionally arousal conditions (27–30). As another vital node for noradrenergic activity modulation, the locus coeruleus (LC) supplies the majority of NE projections to other brain regions (31). Human neuroimaging studies supported the findings from animal studies, and further revealed that noradrenergic activation is associated with an even larger scale of neural networks, as well as a widespread functional changes in NE dynamics (32–35).

The connections between NE and memory have been extensively examined in the past few decades, and several reviews on the neuromodulation of NE in memory have been reported (29, 36–40). However, these reviews contained a relatively small amount of literature, and a comprehensive view of this field in a larger scope is needed. Bibliometrics has become a method with widespread applications in the medical field analysis, and it allows the analysis of vast amounts of publications from public literature databases, at a macroscopic level (41). The quantitative analysis and multicategory visualization of bibliometrics have been significantly highlighted (42–44). Additionally, the trajectory tracing ability of bibliometrics is a brilliant point which enables scholars to explore the elaborate networks of publications in multiple dimensions (45, 46).

Studies related to the interplay between NE and memory have not, as yet, been systematically analyzed from bibliometric perspective. Thus, the objective of the current bibliometric analysis is to fill this gap in the knowledge domain. Our results of the core themes in this area would be valuable guidance for future investigation, and the analysis of global structure would be helpful for scholars in this field to find the core journals and potential collaborators.

## Materials and methods

### Data collection

**Figure 1:**
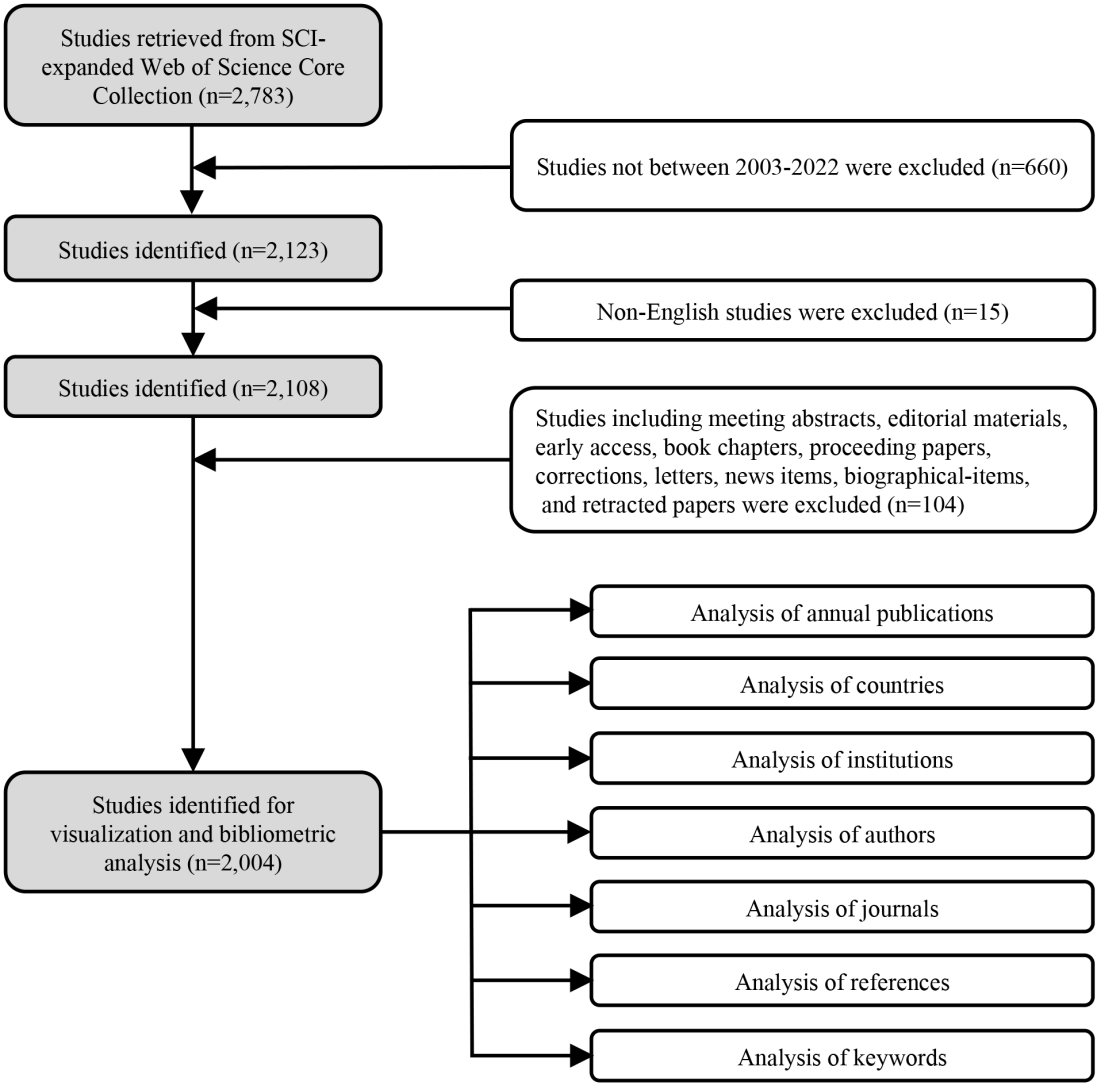
Flowchart depicting the data collection process.

Documents were extracted from the Web of Science Core Collection (WOSCC) database on April 22^nd^ 2023 using the following terms: TS = memory AND TS = (norepinephrine OR noradrenaline) with a time span of 2003-2022. Document types were restricted to articles and reviews, and the language of publication was limited to English. Detailed information on the data collection process is depicted in **Figure 1**.

### Data analysis

In the current study, Microsoft Excel, GraphPad Prism, online bibliometrics platform, CiteSpace, VOSviewer, and R-*bibliometrix*, were employed for bibliometric analysis. Microsoft Excel 2019 was used to create tables, and GraphPad Prism (9.3.1) was applied to generate bar graphs. The bibliometrics website (http://bibliometric.com/) was used to exhibit the global collaboration map considering its advantage in editable and intuitive graphs. CiteSpace (6.2.R2), on the other hand, is a free Java application that offers advantages in the flexibility of data manipulation. In the present study, CiteSpace was employed to layout the keyword citation bursts diagram that revealed the evolution of research emphasis (47). VOSviewer (1.6.18), a standard bibliometric approach based on JAVA, has a potent capability to process and present a substantial amount of bibliometric data (48). In our current study, VOSviewer was applied to exhibit the keyword co-occurrence network and the co-authorship networks of countries, institutions, and authors. The R-*bibliometrix* (4.1.0) is a package under the *R* environment that offers powerful functions in the comprehensive bibliometric analysis of outputs, collaborations, sources, and keywords (49). Furthermore, R-*bibliometrix* offers the possibility to evaluate bibliometric outcome with multiple parameters. For instance, Bradford’s law was utilized to assess the academic influence of journals (50), while *h*-index and *g*-index were two parameters to determine the academic impact of authors (51, 52).

## Results

### Analysis of annual publications

**Figure 2:**
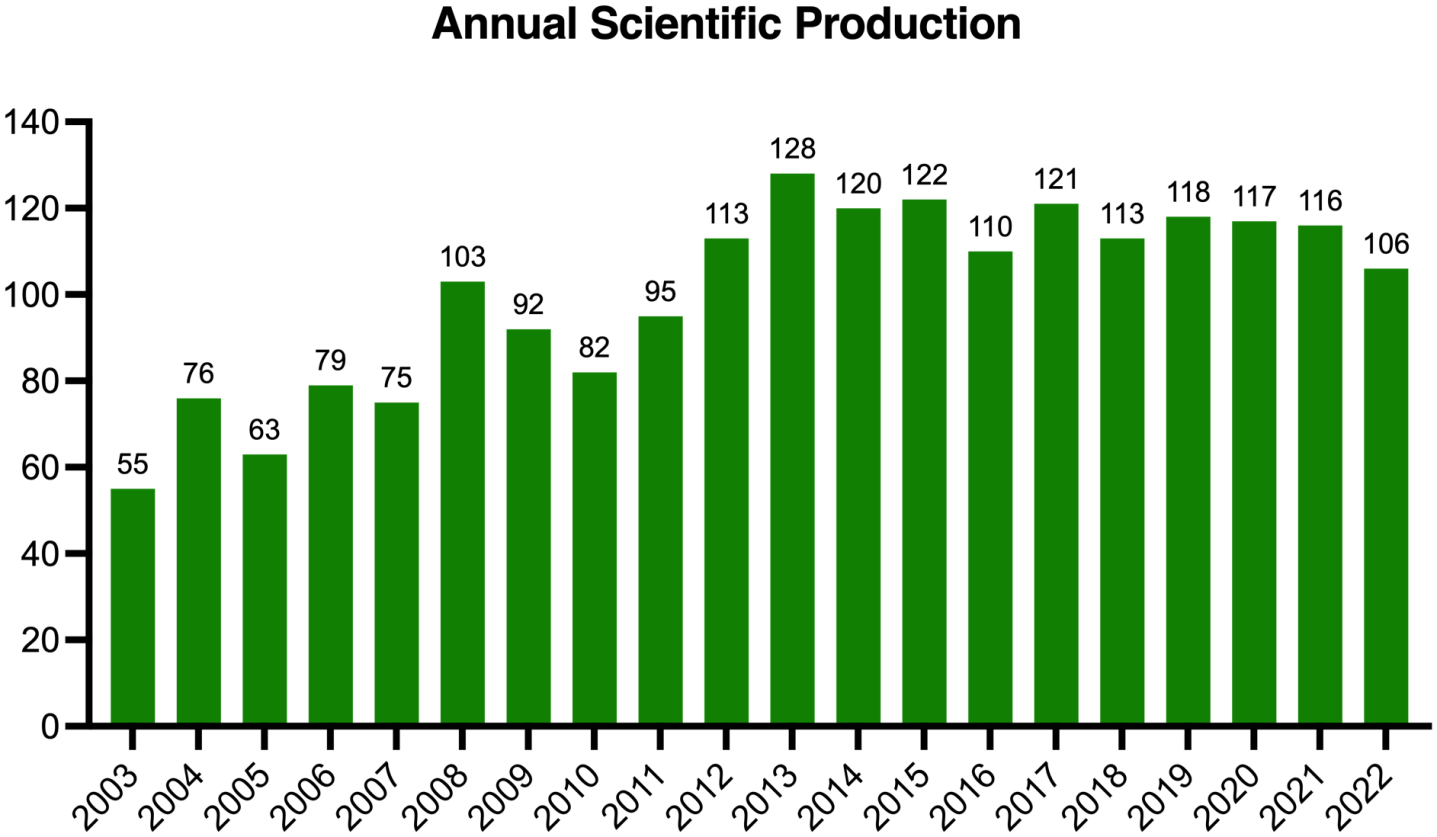
Annual global output from 2003 to 2022.

In total, 2004 studies met the inclusion criteria, and the global output showed a generally upward tendency (**Figure 2**). Research in this field was relatively slow to develop prior to 2008 with fewer than 80 annual publications, and then the annual publication reached a peak of 128 papers in 2013. During 2014-2022, the amount of global output stayed comparatively steady, and fluctuated at approximately 115 papers annually.

### Analysis of countries

**Figure 3:**
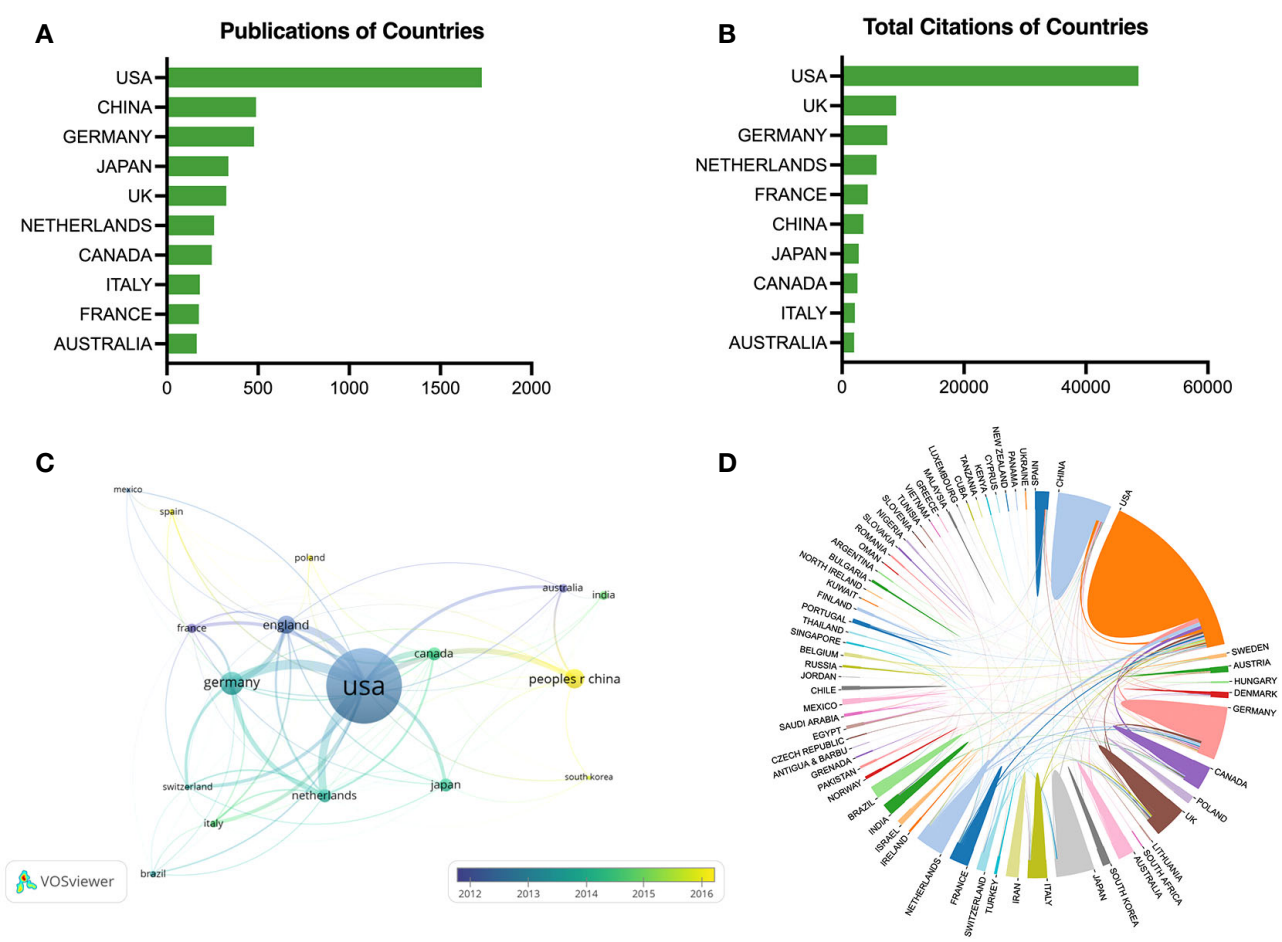
Co-authorship analysis of countries. **(A)** The top ten countries in publications. **(B)** The top ten countries in citations. **(C)** Temporal pattern of country co-authorship network. **(D)** Cooperation map of countries.

The top ten most fruitful countries are displayed in **Figure 3A**, and the leading three countries were the USA (1727 publications), China (489 publications), and Germany (478 publications). **Figure 3B** exhibits the total citations of the leading countries, and the USA ranked first with 48604 citations, followed by the UK (8886 citations), and Germany (7408 citations). The temporal pattern for country co-authorship is depicted in **Figure 3C**, and the threshold of publication was set to 25 for each country. The amount of production of a country was presented by the size of the node, and the active stage was represented by the color gradient. As an example, the node of France was tagged in dark blue color, indicating an early-stage activity for the researchers from France. Poland shown in light yellow indicates that researchers from Poland participated more lately in this field. **Figure 3D** shows the global cooperation networks, USA-Germany (42 collaborations), USA-Netherlands (34 collaborations), and USA-UK (32 collaborations) ranked as the top three among the 263 collaborations worldwide.

### Analysis of institutions

**Figure 4:**
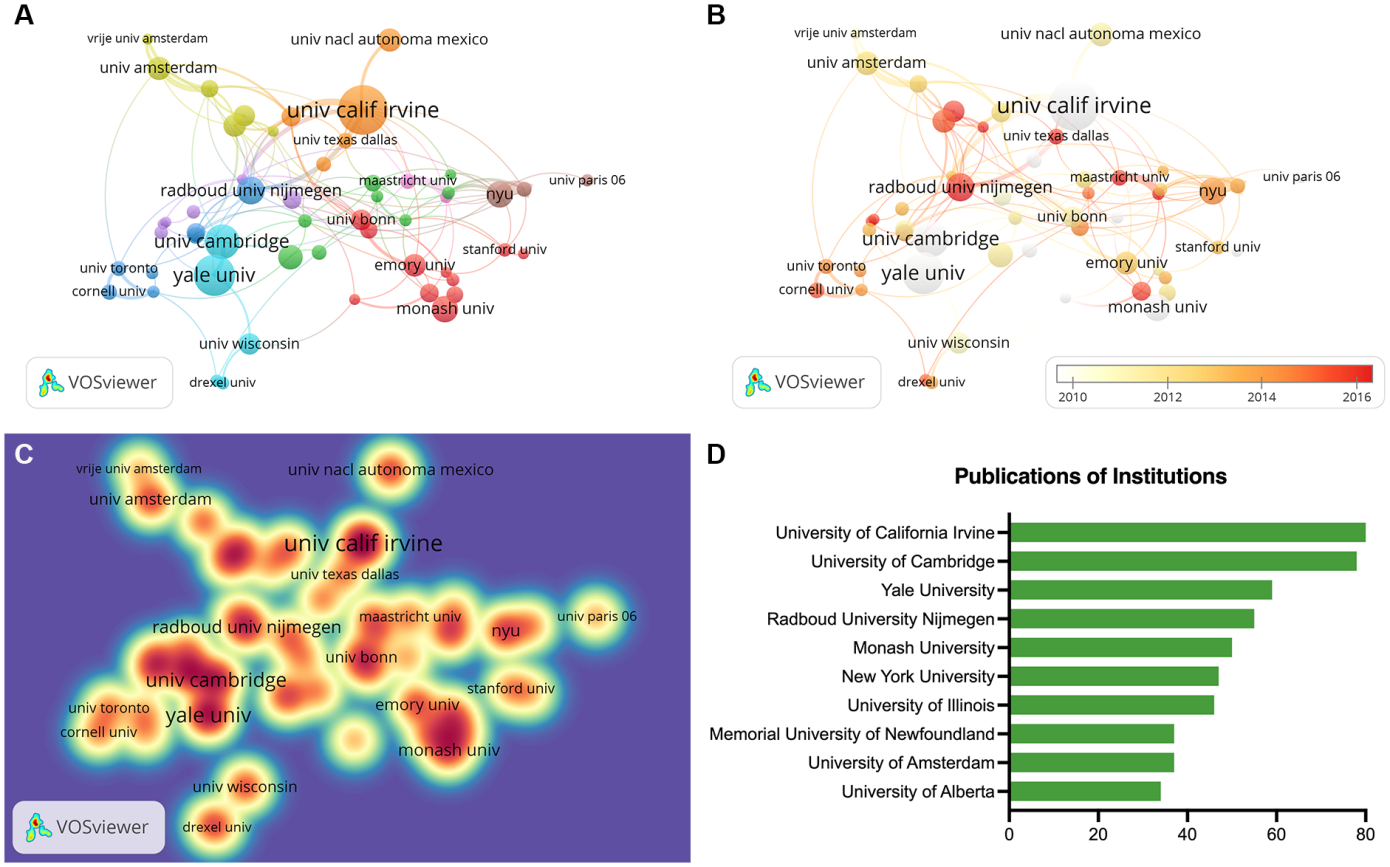
Co-authorship analysis of institutions. **(A)** Cluster visualization of institutions. **(B)** Temporal pattern of institution co-authorship network. **(C)** Frequency visualization of institutions. **(D)** The top ten institutions in publications.

The institution co-authorship networks are displayed in **Figure 4**, and institution with at least 12 publications were selected to create the collaboration networks. **Figure 4A** illustrates the clusters of institutional collaboration networks. University of California Irvine was presented by the largest node, indicating the strongest cooperation level. The thickest connecting line was between University of California Irvine and University of Groningen, indicating the closest collaboration between these two institutions. **Figure 4B** represents the temporal pattern for institutional co-authorship, and the active stage of an institution was depicted according to the color gradient. For instance, University of California Irvine marked in light grey suggests that researchers of this university engaged actively in the initial phase of this topic. Radboud University Nijmegen marked in dark red suggests that scholars from this university were active in recent period. **Figure 4C** depicts the frequency of institutional connections, and University of California Irvine had the highest frequency of cooperation, followed by University of Cambridge and Yale University. **Figure 4D** and **Table 1** display the most influential institutions on this research subject, and the leading three in publications were University of California Irvine (n=80), University of Cambridge (n=78), and Yale University (n=59).

**Table 1:** The top ten most active institutions.

Rank	Institution	Country	Publications	Total citations
1	University of California Irvine	USA	80	1651
2	University of Cambridge	UK	78	842
3	Yale University	USA	59	801
4	Radboud University Nijmegen	Netherlands	55	239
5	Monash University	Australia	50	328
6	New York University	USA	47	529
7	University of Illinois	USA	46	260
8	Memorial University of Newfoundland	Canada	37	355
9	University of Amsterdam	Netherlands	37	295
10	University of Alberta	Canada	34	437

### Analysis of authors

As listed in **Table 2**, Benno Roozendaal (with 35 publications and 5475 total citations) was the most prolific author, followed by James McGaugh (with 30 publications and 5363 total citations) and Amy Arnsten (with 21 publications and 3202 total citations). Moreover, these three researchers also had the leading positions in terms of *h*-index and *g*-index.

**Table 2:** The top ten most productive authors.

Rank	Author	Institution	Publications	Total citations	*h*-index	*g*-index
1	Benno Roozendaal	Radboud University Nijmegen	35	5475	28	35
2	James McGaugh	University of California Irvine	30	5363	27	30
3	Amy Arnsten	Yale University	21	3202	19	21
4	Trevor Robbins	University of Cambridge	21	3738	18	21
5	Carolyn Harley	Memorial University of Newfoundland	20	1258	17	20
6	Marie Gibbs	Monash University	19	760	15	19
7	Babara Sahakian	University of Cambridge	14	1992	14	14
8	Mara Mather	University of Southern California	15	959	13	15
9	Craig Berridge	University of Wisconsin-Madison	13	2742	12	13
10	Oliver Wolf	Ruhr University Bochum	14	1384	12	14

**Figure 5:**
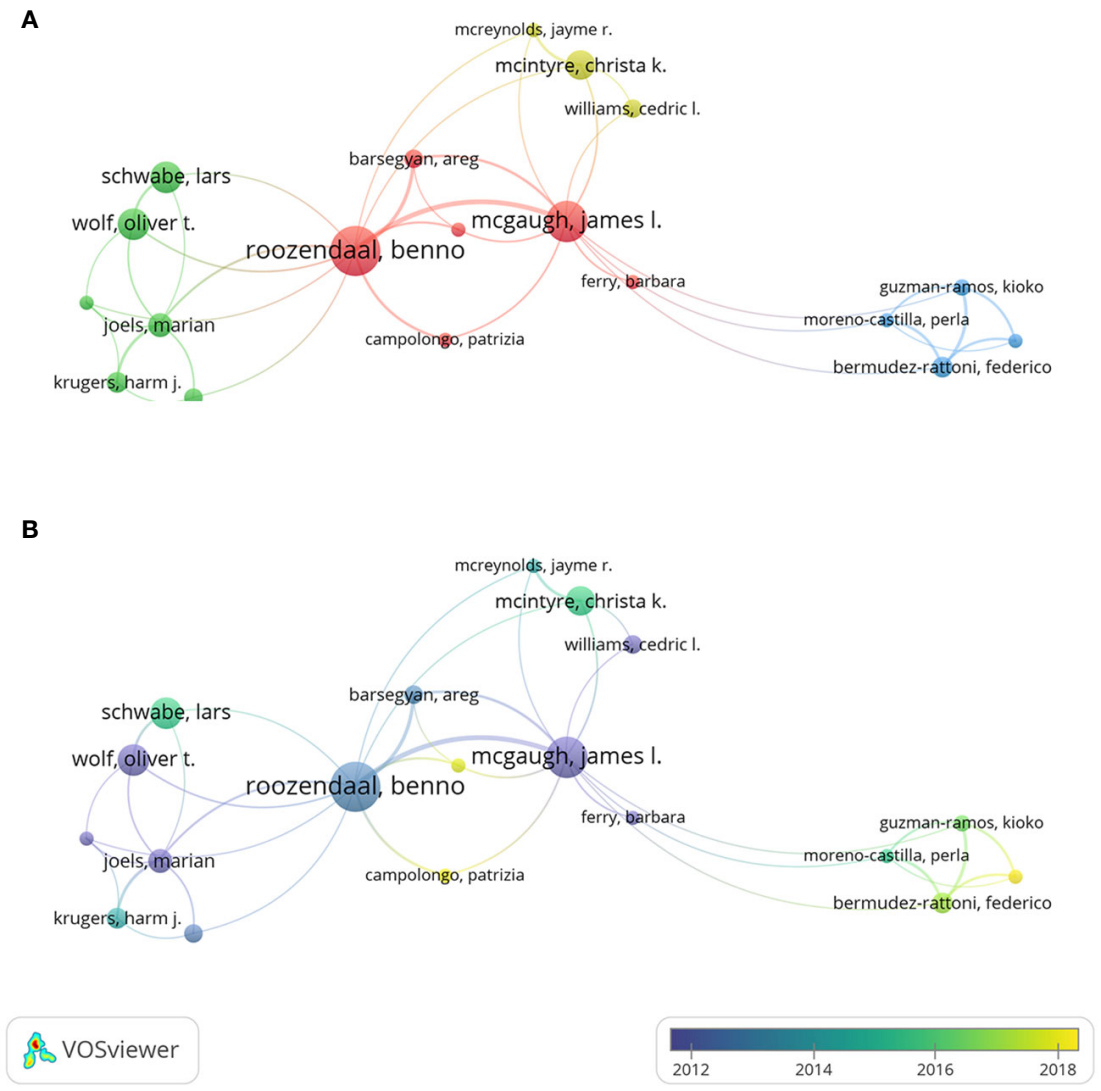
Co-authorship analysis of authors. **(A)** Cluster visualization of authors. **(B)** Temporal pattern of author co-authorship network.

**Figure 5** exhibits the co-authorship networks of author, and the minimal publication of an author was set to 5 for the readability of the collaboration network. **Figure 5A** shows the clusters of co-authorship network by the frequency of collaborations between each author. Benno Roozendaal had a total link strength of 37, indicating a strong cooperation with others. The thickest connecting line was between Benno Roozendaal and James McGaugh, indicating the closest collaboration between these two scholars. The temporal pattern of co-authorship network is displayed in **Figure 5B**. For instance, Marian Joëls from University of Groningen was labeled in dark blue color, suggesting that this researcher engaged more frequently in the early-stage on this theme. Daniel Osorio-Gómez from National Autonomous University of Mexico was labeled in light yellow color, suggesting a more recent academic activity for this researcher.

### Analysis of journals

Among the most relevant journals, *Neurobiology of Learning and Memory* had the largest number of outputs (n=75), followed by *Neuroscience* (n=74) and *Journal of Neuroscience* (n=71). From the perspective of total citations, the foremost journals were *Journal of Neuroscience* (n=764), *Learning & Memory* (n=509), and *Neurobiology of Learning and Memory* (n=495). According to the impact factor (IF) 2022, the top three journals were *Neuropsychopharmacology* (IF=8.294), *Journal of Neuroscience* (IF=6.709), and *Neuropharmacology* (IF=5.273) (**Table 3**). These data would be helpful for scientists in finding the core journals on this subject.

**Table 3:** The top ten most relevant journals.

Rank	Journal	Publications	Total citations	IF (2022)
1	*Neurobiology of Learning and Memory*	75	495	3.109
2	*Neuroscience*	74	413	3.708
3	*Journal of Neuroscience*	71	764	6.709
4	*Behavioural Brain Research*	65	142	3.352
5	*Psychopharmacology*	61	333	4.415
6	*Neuropsychopharmacology*	51	252	8.294
7	*Learning & Memory*	49	509	2.699
8	*Brain Research*	38	156	3.610
9	*Neuropharmacology*	35	84	5.273
10	*PLoS One*	34	40	3.752

### Analysis of highly-cited documents

The level of academic impact in similar fields and all fields were evaluated by “local citation” and “global citation”, respectively (53). As listed in **Table 4**, the study published in *Brain Research Reviews* titled “The locus coeruleus-noradrenergic system: modulation of behavioral state and state-dependent cognitive processes” had the highest local citation and global citation (219 local citations and 1679 global citations). This highly-cited publication by Berridge and Waterhouse systematically introduced the locus coeruleus-norepinephrine (LC-NE) system, ranging from the anatomical organization to the functions of modulation on synaptic efficacy, neuronal dynamics, and cognition. As the LC-NE system supplies NE throughout the central nervous system via the widespread efferent projections, this review comprehensively formed the foundation for the involvement of NE in memory, and provided promising potential targets for the strategies of cognitive disorders (54).

**Table 4:** The top ten highly-cited documents.

Rank	Document	DOI	Local citations	Global citations
1	Berridge CW, 2003, *Brain Res Rev*	10.1016/s0165-0173(03)00143-7	219	1679
2	Sara SJ, 2009, *Nat Rev Neurosci*	10.1038/nrn2573	182	1030
3	McGaugh JL, 2004, *Annu Rev Neurosci*	10.1146/annurev.neuro.27.070203.144157	132	1561
4	Murchison CF, 2004, *Cell*	10.1016/s0092-8674(04)00259-4	119	292
5	Ramos BP, 2007, *Pharmacol Therapeut*	10.1016/j.pharmthera.2006.11.006	89	450
6	Roozendaal B, 2006, *P Natl Acad Sci USA*	10.1073/pnas.0601874103	78	368
7	Hu HL, 2007, *Cell*	10.1016/j.cell.2007.09.017	66	360
8	Lalumiere RT, 2003, *J Neurosci*	10.1523/jneurosci.23-17-06754.2003	62	155
9	Roozendaal B, 2009, *Nat Rev Neurosci*	10.1038/nrn2651	62	1148
10	Sara SJ, 2012, *Neuron*	10.1016/j.neuron.2012.09.011	62	525

### Analysis of keywords

**Figure 6:**
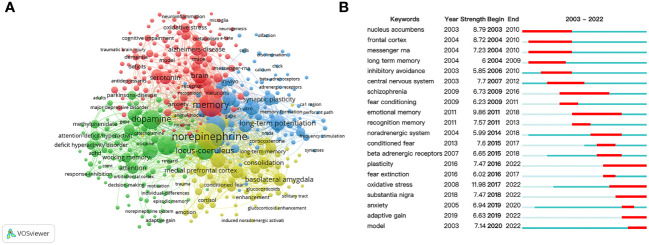
Co-occurrence analysis of keywords. **(A)** Cluster visualization of keywords. **(B)** Citation bursts of the top twenty keywords.

The keyword co-occurrence networks are demonstrated in **Figure 6**, and keywords with a minimal occurrence of 10 were used for visualization.

A total of 4 clusters were organized as presented in **Figure 6A**. Cluster 1 concentrated on the memory-enhancing effect of stress hormones, and its main nodes were “norepinephrine”, “basolateral amygdala”, “medial prefrontal cortex”, “consolidation”, “long term memory”, “enhancement”, “cortisol”, and “glucocorticoids”. Cluster 2 mainly focused on memory-related diseases, and the main nodes were “memory”, “anxiety”, “neurons”, “antidepressant”, “Alzheimer disease”, “dementia”, “deficits”, and “cognitive impairment”. Cluster 3 highlighted the importance of DA in different learning experiences, and the main nodes were “dopamine”, “prefrontal cortex”, “locus coeruleus”, “working memory”, “attention”, “ADHD”, “response-inhibiting”, “decision-making”, and “reward”. Cluster 4 mainly focused on NE modulation of synaptic plasticity, and the main nodes were “noradrenaline”, “hippocampus”, “long-term potentiation”, “modulation”, “*in-vivo*”, “synaptic plasticity”, “dentate gyrus”, and “noradrenergic modulation”.

The keyword citation bursts are shown in **Figure 6B**, the active period and the interval of the citation bursts are represented by blue and red bars. The keywords were organized into three stages. In the first stage (2003–2008), keywords were “nucleus accumbens”, “frontal cortex”, “messenger RNA”, “long term memory”, “inhibitory avoidance”, and “central nervous system”. During 2009-2015, the research hotspots shifted to “schizophrenia”, “fear conditioning”, “emotional memory”, “recognition memory”, “noradrenergic system”, “conditioned fear”, and “beta adrenergic receptors”. In recent years (2016–2022), “plasticity”, “fear extinction”, “oxidative stress”, “substantia nigra”, “anxiety”, “adaptive gain”, and “model” became the main research frontiers.

## Discussion

### General knowledge framework

The annual scientific productivity is indicative of the development trends of a certain research topic (55, 56). In the current study, we found that the annual output of publications showed a fluctuating but generally upward tendency. Specifically, the publication number first displayed a rising trend prior to 2014 (average rate of growth: 7.35%), suggesting that this theme received increasing attention. One underlying reason might be that some influential reviews paved the foundation and highlighted the promising prospects of this field, which caused a minor burst in 2013 (20, 57, 58). Another factor might relate to the emergence of optogenetics in 2011, which led to a new era in neuroscience and provided a significant breakthrough for subsequent research (59–61). Finally, major events in brain science from 2005 to 2014 (Switzerland: Blue Brain Project, 2005; European Union: Human Brain Project, 2013; USA: BRAIN Initiative, 2013) might also greatly promote the development of this field. Afterwards, annual scientific productivity demonstrated a slightly decreasing pattern during 2014-2022 (average rate of growth: -1.54%). This might suggest that researchers encountered some bottlenecks in this field, and require breakthroughs in the upcoming explorations. Nevertheless, the overall growing trend in global output revealed that this research theme is receiving continuous attention worldwide.

Our results of the national distribution revealed the deepest academic accumulation of the USA regarding scientific output and global cooperation. For example, the USA had more than three times the number of publication (n=1727) than the country ranked second on the list (China, n=489). The total citations of the USA (n=48604) were over five times than those of the country ranked second (UK, n=8886). According to the institution ranking in **Table 1**, four of the most productive institutions (University of California Irvine, Yale University, New York University, and University of Illinois) were located in the USA. Notably, close collaboration clusters were observed from the institutional co-authorship networks, such as University of Bonn, Emory University, University of Hamburg and Stanford University; University of California Irvine, Radboud University Nijmegen, University of Amsterdam, and Maastricht University; Yale University, University of Cambridge, and University of Bristol. Interestingly, these findings are in line with the top three rankings of international collaborations (USA-Germany, USA-Netherlands, and USA-UK) in **Figure 3D**. Together, we envision that the global collaboration network in this area will expand further, and the USA is still taking an absolute lead in this research area.

The academic impact of a scholar can be reflected by *h*-index and *g*-index (51, 52). As shown in **Table 2**, Benno Roozendaal and James McGaugh had the leading positions in *h*-index and *g*-index. Moreover, Benno Roozendaal and James McGaugh were the only two scholars with more than 30 publications and more than 5000 total citations, which reflected their significant academic accumulations. From the perspective of author collaborative relationship, Benno Roozendaal (Radboud University Nijmegen, Netherlands), James McGaugh (University of California Irvine, USA), Lars Schwabe (University of Hamburg, Germany), Oliver Wolf (University of Bochum, Germany), and Christa McIntyre (University of Texas, USA) were the most active authors in this field, and they have formed close collaborative networks with each other (**Figure 5A**). James McGaugh is a pioneer in the investigation of memory processes underlying the effects of stress hormones. He devoted himself to research on emotional arousal as well as the function of the amygdala, and has produced impactful articles with Benno Roozendaal, Federico Bermudez-Rattoni, Christa McIntyre, and Jayme McReynolds (14, 19, 28, 62–64). Benno Roozendaal is another most important researcher in this field with the greatest number of publications and citations on this subject. He made fruitful achievements and gained a deep understanding of the interplay between stress, memory, and the amygdala (57). He recently discovered novel directions on memory quality and its underlying neural circuits, via the collaborations with Lars Schwabe, Patrizia Campolongo, and Erika Atucha (32, 65–67). In summary, the results showed that these key researchers had great impact and in-depth understandings of this research area, their significant international and/or institutional contributions drove the rapid development of this field.

### Research hotspots

Keyword co-occurrence analysis has been commonly applied to reflect the research hotspots and core contents of the literature (68). In **Figure 6A**, the hotspots in this field were distributed into several independent, yet closely related, clusters. The evolution of research on this topic could be organized into four subthemes according to the keyword clusters.

The yellow cluster focused on the memory-enhancing effect of stress hormones and the main brain regions involved. Extensive evidence has pointed out the central role of NE in regulating other stress hormones under emotional memories, and the amygdala plays a pivotal role under emotionally arousal conditions (27–30). Other brain regions, namely hippocampus and prefrontal cortex, also play critical roles during stress-related memory and have close interactions with the amygdala. It was reported that NE administration into the amygdala, hippocampus, or prefrontal cortex induced enhanced emotional memory under stress (36, 57, 69, 70). Notably, such NE activation enhances not only high-arousal experiences such as fear memory, but also low-arousal experiences such as recognition memory (66). Thus, the effect of NE on a specific type of memory has become a promising direction that is worth studying.

The red cluster focused on cognitive disorders, including PTSD, Alzheimer’s disease, and depression, which are closely related to aberrant memory processes (3–10, 71). Interestingly, it is commonly found that long-lasting but less specific negative memory is thought to be the main risk factor of cognitive disorders (6, 11, 12). For example, it has been shown that enhanced memory with reduced accuracy lies at the center of PTSD (6). Although recent findings showed that NE can enhance the duration of memory while retaining the memory specificity, the underlying cellular and network mechanisms remain completely unknown (27, 30). Therefore, it would be especially interesting to uncover the underlying mystery of memory specificity in the future investigations.

The green cluster revealed the strong connections between NE and DA, two crucial modulators controlling memory processes. In earlier studies, Takeuchi et al. applied optogenetic manipulations in a mouse model and discovered that LC neurons expressing tyrosine hydroxylase regulate memory enhancement with a concurrent hippocampal activation of DA (22). Kempadoo et al. also used optogenetics and found that DA activation in the dorsal hippocampus enhances spatial memory (72). More recently, it was reported that the LC can broadcast simultaneous release of NE and DA throughout the brain, and DA-NA interactions during memory processes were mainly studied in the hippocampus and prefrontal cortex (73–77). Thus, more focus on the effective interventions of both NE and DA would be possible targets for the therapeutics of various disorders with memory deficits.

The blue cluster mainly focused on noradrenergic modulation and synaptic plasticity. It is believed that synaptic strength changes are the core of memory formation, and the ability of NE to regulate synaptic plasticity is an essential mechanism of memory regulation (78). Several studies have demonstrated that NE activates both presynaptic and postsynaptic adrenergic receptors in different specific neural circuitries (79–82). Importantly, it is noticeable that the emerging technical advances in imaging, such as fiber photometry and calcium imaging, have provided wider research perspectives than the current pharmacological methodology (83, 84). Hence, future investigations should emphasize more on these techniques for a deeper interpretation of NE regulation on synaptic plasticity.

Furthermore, from **Figure 6B** depicting the citation bursts of keyword, we found not only the shifting of research hotspots, but also the transformation of paradigms. Particularly, the earlier focus on the molecular level, single brain region, or single neurons, has switched to a systems level, such as neural circuits. Such deeper understanding of brain function might benefit from the explosion of new technologies that enable us to manipulate neural circuits more precisely and rapidly. Another switch of research hotspot, from central nervous system to intuitive behavioral readout, would further heighten our understanding of the circuits underlying modulation activities.

### Outlook

Several aspects should be noted for the future investigations in this research field. First, since a single memory process includes multiple cellular mechanisms and large-scale networks, research efforts should be focused on the discrete aspects of a single type of memory, such as the longevity, accuracy, or specificity, of fear memory or recognition memory. Second, it remains unknown whether the memory-enhancing effect of NE was conducted by excitatory or inhibitory subpopulations of neurons, or different layers of neurons. In recent rodent studies, the application of optogenetics has allowed the examination of a specific layer in multiple memory processes (85–87). Therefore, future explorations should investigate the role of neurons in a more detailed manner. Third, researchers should pay more attention to the robust tools that could enable us to delve into the circuit dynamics in a deeper dimension. For instance, fiber photometry has allowed the real-time observation of specified pathways in memory (84). Moreover, a recently developed *in vivo* NE sensor has overcame the limitation in the transient NE activity measurement, which brought us a technical breakthrough in the examination of precise and rapid NE dynamics (88). Finally, regarding methodology in data mining, a novel strategy termed triangulation could be considered for more precise tracking of the timely research frontiers from the rapidly growing databases. Such triangulation method integrates text mining, machine learning, and bibliometric mapping, and greatly improved the capability of qualitative and quantitative analysis in bibliometrics (56, 89–92).

## Conclusion

Our study revealed the research pattern and development trends of literature related to the connection between NE and memory via bibliometric analysis. The general global trend on this topic showed an ascending trend over the past two decades. The USA had the deepest academic accumulation regarding scientific outputs and global cooperation. Meanwhile, University of California Irvine from the USA was the most fruitful institution. Benno Roozendaal and James McGaugh were the most prolific scholars in this research domain. The research emphasis of NE has shifted from the molecular level or single brain region to the investigation of neural circuits. In summary, our results of the knowledge mapping would provide valuable insights into the global research landscape.

## Limitations

Our current study has some limitations that are worth noting. First, publications were only retrieved from WOSCC, which might cause the omission of potential data from other sources, such as PubMed and Scopus. In addition, we only extracted the published studies during 2003-2022. Although the time span of 20 years was adequate to reflect the research trends, some of the original papers that formed the basis of this field may be missing. Finally, we only collected the publications written in English, therefore, some relevant non-English studies were not covered.

## Data availability statement

The original contributions presented in the study are included in the article/supplementary material. Further inquiries can be directed to the corresponding author.

## Author contributions

YT designed the framework of the paper. QS collected the data. QS generated the figures. YT drafted the manuscript. All authors contributed to the article and approved the submitted version.
